# Abiraterone-Induced Hypokalemia: A Case Report

**DOI:** 10.7759/cureus.42533

**Published:** 2023-07-27

**Authors:** Bernard I Nkwocha, Meenu Singh

**Affiliations:** 1 Department of Internal Medicine, University of Utah School of Medicine, Salt Lake City, USA

**Keywords:** urologic oncology, hypokalemia, abiraterone-induced hypokalemia, electrolyte imbalance, androgen-deprivation therapy, abiraterone, prostate cancer (pca), castration-resistant metastatic prostate cancer

## Abstract

Abiraterone, an androgen biosynthesis inhibitor drug approved by the Food and Drug Administration (FDA) in 2011 for the treatment of metastatic prostate cancer, has seen an increase in prescriptions over the years, owing largely to the aging population and the association of prostate cancer with increasing age. As the rate of abiraterone prescription increases, it is important for physicians to be aware of its adverse effects profile to improve patient outcomes. This case report explains the mechanism, clinical presentation, and management of abiraterone-induced hypokalemia in a 67-year-old male with prostate cancer and highlights the importance of close monitoring and management of electrolyte levels for patients on abiraterone.

## Introduction

Prostate cancer is the most commonly diagnosed cancer among males in the United States of America. It is predicted that there will be approximately 288,300 new cases of prostate cancer reported in 2023, which is approximately 800 cases daily. Prostate cancer is also recognized as the second most common cause of cancer-related mortality among men in the United States, with an estimated 34,700 deaths in the year 2023 [1]. Approximately one in every eight men in the United States is estimated to receive a diagnosis of prostate cancer at some point in their lifetime. Furthermore, the likelihood of being diagnosed with prostate cancer increases with age, ranging from approximately one in every 456 men under the age of 50 to one in every 11 men aged 70 and older being affected [2].

Prostate cancer relies on androgens, particularly testosterone, for its growth, making androgen deprivation therapy (ADT) the cornerstone of treatment. Androgen deprivation can be achieved through medical or surgical methods. Medical ADT relies on Gonadotropin-releasing hormone (GnRH) agonists (e.g., leuprolide) and GnRH antagonists (e.g., degarelix) that act on the anterior pituitary gland and inhibit the release of gonadotropins, reducing testosterone production. GnRH agonists stimulate the receptors initially but eventually desensitize them while GnRH antagonists directly block the receptors. Surgical ADT involves the surgical removal of bilateral testes (orchidectomy). Both medical and surgical approaches are effective in reducing androgen levels (castration), depriving the cancer cells of the necessary hormones for growth. However, due to the reversibility of treatment, comparable efficacy, and less psychological and body image impact, medical therapy has become the treatment of choice for ADT in metastatic prostate cancer [3-5]. The goal is to achieve castrate levels of serum testosterone (<50 ng/dL).

Certain forms of both early-stage and late-stage prostate cancer exhibit resistance to standard ADT treatments, prompting the exploration of alternative options. These alternatives include chemotherapeutic drugs, androgen receptor antagonists, such as flutamide or enzalutamide, and medications targeting the androgen synthesis pathway. Androgen synthesis pathway drugs play a crucial role in the treatment of castration-resistant prostate cancer (CRPC), as certain tumor cells develop the ability to produce androgens locally within the prostate tumor microenvironment, bypassing the need for adrenal and testicular androgens. Abiraterone acetate, one such drug, is a 3β-sterol agent that works by irreversibly inhibiting enzymes (17-20 lyase and 17 alpha-hydroxylase) in the adrenal gland, testes and prostate cancer cells (local and metastasized) [6]. These enzymes are needed to produce glucocorticoids (cortisol) and androgens. The reduction in glucocorticoids in the adrenal gland decreases the negative feedback at the anterior pituitary that typically blocks adrenocorticotropic hormone (ACTH) release. Increased ACTH secretion in the background of reduced/blocked glucocorticoids and androgens synthesis leads to excess mineralocorticoid production from the adrenal gland, causing edema, hypertension, and hypokalemia. Hypokalemia occurs in 17-30%, hypertension in 7-37%, and fluid retention in 25-27% of patients taking abiraterone [7]. 11-deoxycorticosterone, commonly called deoxycorticosterone, is the major mineralocorticoid implicated.

Since its approval in 2011 by the Food and Drug Administration (FDA) for castration-resistant prostate cancer, the prescription rate of abiraterone has been steadily rising; from 71,423 in 2013 to 100,371 in 2015 [8]. This case report explores electrolyte derangements, specifically low potassium levels, and associated morbidity in patients undergoing abiraterone treatment for prostate cancer. The findings contribute to a better understanding of the challenges involved in abiraterone therapy and underscore the need for vigilant monitoring and proactive intervention.

## Case presentation

The patient, a 67-year-old man, with a history of prostate cancer of five years duration with recent small cell transformation, extensive bony metastatic disease, and recent surgical intervention for right hip pathologic fracture, presented with profound muscle weakness, hypoxic respiratory failure, confusion, severe hypokalemia, and failure to thrive. The patient had received care in a different state until this presentation.

The patient had been intermittently treated with leuprolide, abiraterone, and prednisone for five years, with more consistent use for several months prior to the index admission. During this time, he was noted to have intermittent low serum potassium levels ranging from 2.4 to 3.3 mmol/L along with an episode of hospitalization for new-onset atrial fibrillation with rapid ventricular response presumed to be from hypokalemia of unclear cause. This led to treatment with amiodarone 200 mg daily, potassium 40 meq daily, spironolactone 100 mg twice daily, and magnesium 400 mg twice daily until present admission. The patient was also given chlorthalidone 25 mg daily, secondary to fluid retention in the form of bilateral lower extremity edema and pleural effusions.

During the index hospitalization, the patient was unable to keep his head raised without support. The patient’s wife said that he had been "wasting potassium" for several months with workups yielding no apparent reason. The Karnofsky Performance Scale (KPS) rating was 30% (range 0 (dead) - 100 (normal)) and the Eastern Cooperative Oncology Group (ECOG) score was 3 (range 0-5, with a score of 5 being the worst). The vital signs at presentation were temperature 36.3°C; BP 96/54 mmHg; pulse rate 105 beats per minute; respiratory rate 16 cycles per minute; and oxygen saturation 97% on 4 L oxygen by nasal cannula. Physical examination was notable for Glasgow Coma Scale of 12 (normal 15), prolonged capillary refill time of more than 3 seconds (normal ≤2 seconds), body mass index of 30 kg/m^2^ (normal range 18.5 - 24.9 kg/m^2^), 3+ bilateral pitting pedal edema, reduced breath sounds bilaterally, an irregular heart rhythm, moderate abdominal distension. Labs were significant for potassium 2.2 mmol/L (normal range 3.5 - 5 mmol/L), sodium 143 mmol/L (normal range 136 - 145 mmol/L), blood urea nitrogen (BUN) 16 mg/dL (normal range <20 mg/dL), creatinine 0.38 mg/dL (normal range 0.7 - 1.3 mg/dL), total bilirubin 1.4 mg/dL (normal range 0.1 - 1.2 mg/dL), and alanine transaminase (ALT) 82 U/L (normal range 5 - 60 U/L). Brain natriuretic peptide (BNP) was 213 pg/mL (normal range < 100 pg/mL). Urinalysis was negative for proteinuria. ECG showed sinus tachycardia with premature ventricular complexes, and the echocardiogram was unremarkable. MRI brain at presentation revealed diffuse parenchymal volume loss with no signs of brain parenchymal metastasis. See Table 1 for a summary of the patient’s presenting vital signs, lab values, and pertinent physical examination findings.

**Table 1:** Patient’s presenting vital signs, lab results, and pertinent physical examination findings *: critically low; ^ɸ^: hypokalemia with fractional excretion of potassium between 1.5% and 6.4% suggests extrarenal causes and 9.5% and 24% suggests renal losses [9] ACTH: adrenocorticotropic hormone; ALT: alanine transaminase; BNP: brain natriuretic peptide; BP: blood pressure; bpm: beats per minute; BUN: blood urea nitrogen; cpm: cycles per minute; K^+^: potassium; Na^+^: sodium; NC: nasal cannula; O2: oxygen; P: pulse rate; R: respiratory rate; T: temperature

Vital signs	Pertinent physical examination findings	Lab results	Lab reference range
T: 36.3⁰C (97⁰F)	General physical: GCS of 12; BMI of 30 kg/m^2^	K^+^: 2.2 mmol/L *	3.5-5mmol/L
P: 105 bpm	Cardiovascular: capillary refill time > 3 seconds; irregular heart rhythm	Na^+^: 143 mmol/L	136-145 mmol/L
BP: 96/54 mmHg	Chest: reduced breath sounds bilaterally	BUN: 16 mg/dL	<20 mg/dL
R: 16cpm	Abdomen: abdominal distension; fluid thrill on percussion	Creatinine: 0.38 mg/dL	0.7 – 1.3 mg/dL
O_2_ saturation: 97% on 4L O_2_ by NC		Total bilirubin: 1.4 mg/dL	0.1 – 1.2 mg/dL
		ALT: 82 U/L	5 – 60 U/L
		BNP: 213 pg/mL ()	<100 pg/mL
		Urinalysis: negative for proteinuria	
		ACTH: 437pg/mL	7.2 – 63.3 pg/mL
		Aldosterone: 5.6 ng/dL	<16/31 ng/dL
		Renin: 2.5 ng/mL/hr	0.2 – 4 ng/mL/hr
		Aldosterone-to-renin ratio: 2.2	<25
		Random urine K^+^: 85 mmol/L	<20 mmol/L
		Fractional excretion of K^+^: 10.7%	See footnote^ ɸ^

During his hospital admission, the patient was treated by a multidisciplinary team that included a nephrologist and an oncologist. Abiraterone, chlorthalidone, and prednisone were held on admission for index hospitalization due to severe hypokalemia. Spironolactone was reduced to 50 mg twice daily secondary to borderline low blood pressure. Potassium supplementation of approximately 120 meq was given with minimal improvement in potassium levels. The patient was started on dexamethasone 4 mg twice a day due to moderate to severe spinal canal stenosis at L4 and L5 from circumferential epidural tumor involvement and lower extremity weakness. Following a 24-hour hold of spironolactone and supplemental potassium to minimize the effect of potential interference with the aldosterone-to-renin ratio and urine potassium results, lab workup revealed ACTH was significantly elevated at 437 pg/mL (normal range 7.2 - 63.3pg/mL), random urine potassium was 85 mmol/L (normal range <20 mmol/L) with fractional excretion of potassium calculated to be 10.7% (9.5-24% indicates renal losses [9]). Aldosterone was 5.6 ng/dL (normal range <16 ng/dL), renin was 2.5 ng/mL/hr (normal range 0.2-4 ng/ml/hr), and aldosterone-to-renin ratio was 2.2 (normal range <25). Based on this workup and the mechanism of action of abiraterone, the patient was diagnosed with abiraterone-induced hypokalemia from mineralocorticoid excess.

A few days after stopping abiraterone and starting dexamethasone, the patient’s potassium supplementation needs improved significantly. The patient was eventually transitioned to hospice care secondary to advanced disease, poor overall response to supportive care, and continuing decline.

## Discussion

Hypokalemia is defined as serum potassium of less than 3.5 mmol/L and is classified in severity based on lab values and the absence or presence of symptoms. Mild hypokalemia is serum potassium of 3.0-3.5 mmol/L; patients can be asymptomatic or have mild symptoms. Moderate hypokalemia is serum potassium of 2.5-2.9mmol/L; It can present with muscle cramps, muscle weakness, arrhythmias and palpitations, constipation, and polyuria. Severe hypokalemia is serum potassium of less than 2.5mmol/L; symptoms include severe muscle weakness, paralysis, fatigue, acute respiratory failure, life-threatening arrhythmias, severe thirst and dehydration, polyuria, constipation/bowel obstruction, paresthesia, confusion, etc.

The patient’s extreme fatigue, arrhythmia, respiratory failure, and confusion were most likely caused by severe hypokalemia, secondary to abiraterone pronounced in the setting of poor oral intake and chlorthalidone use. The patient’s prednisone use of 5 mg/daily was ruled out as a contributor to hypokalemia with elevated ACTH. Given that hypokalemia with fractional excretion of potassium between 1.5% and 6.4% suggests extrarenal causes, and values of 9.5-24% show renal losses [9], this patient's urine potassium level and fractional excretion of potassium (85 mmol/L and 10.7%, respectively) indicated renal losses. The normal aldosterone-to-renin ratio ruled out hyperaldosteronism. Excessive renal potassium loss, likely caused by elevated deoxycorticosterone due to abiraterone, was identified as the primary cause based on the workup.

**Figure 1:**
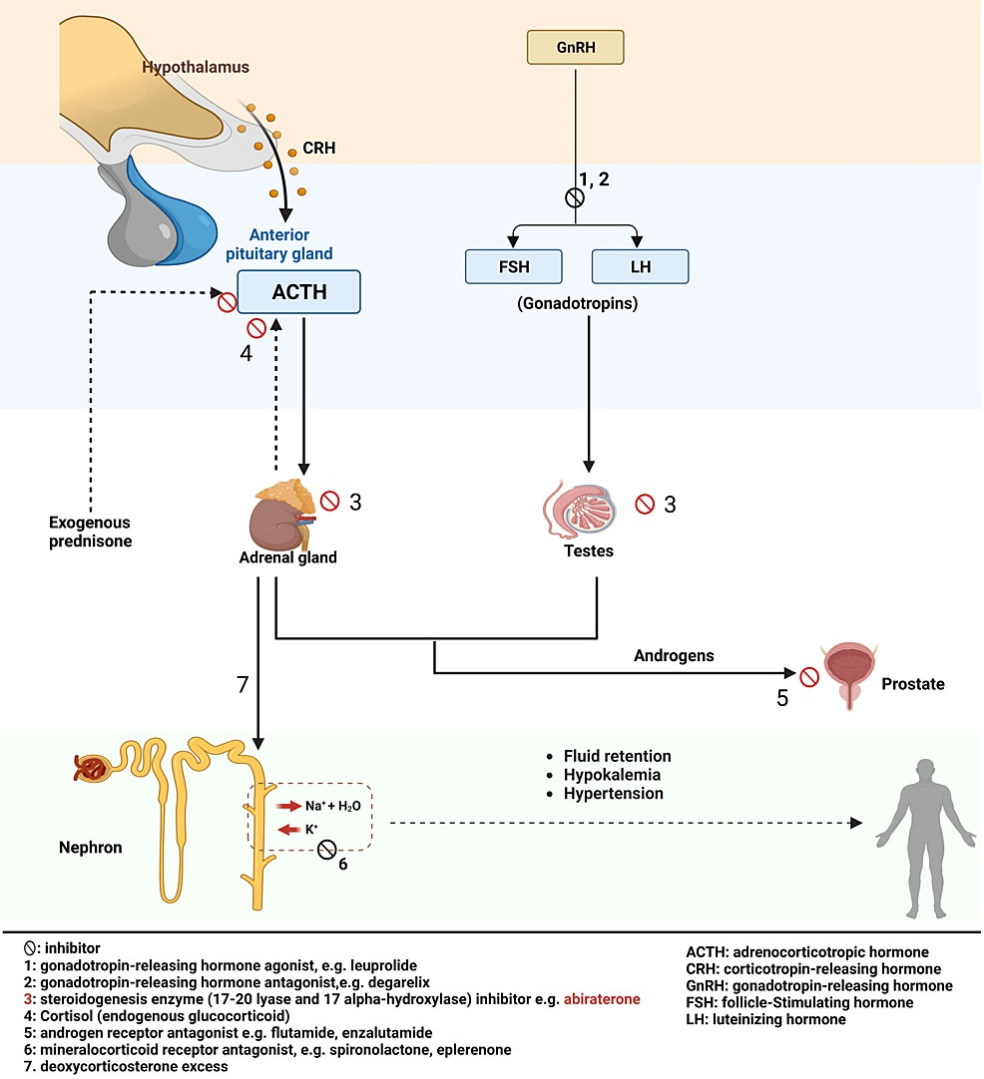
Hypothalamic-pituitary-adrenal axis and mechanism of action of abiraterone and prednisone Created with BioRender.com

**Figure 2:**
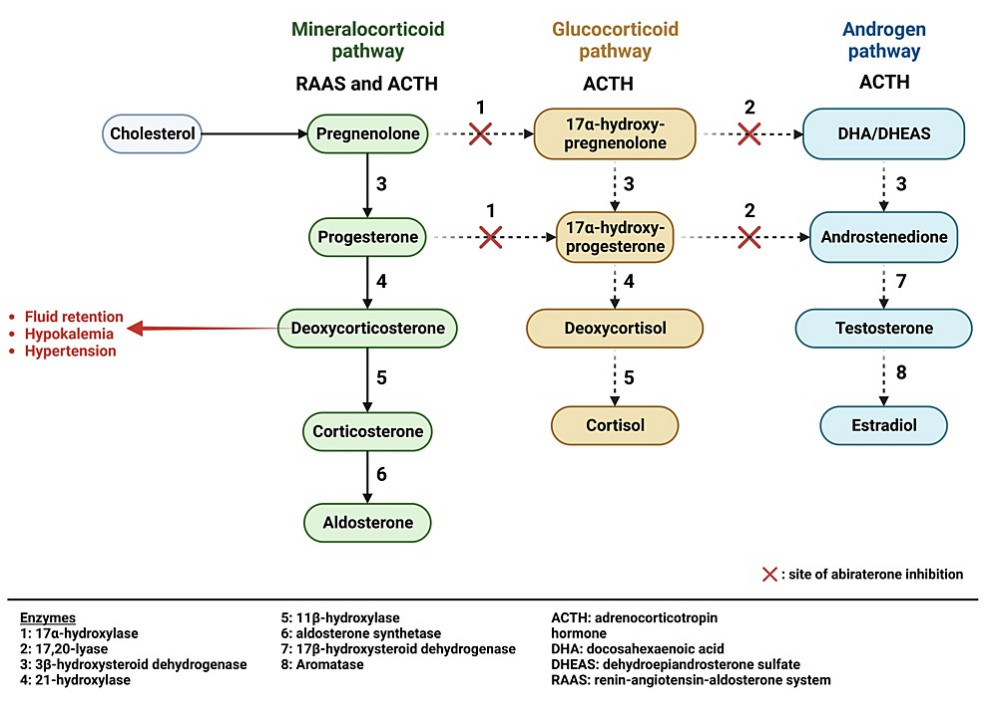
Steroidogenesis pathway in the adrenal gland Created with BioRender.com

Abiraterone-induced hypokalemia is a known adverse effect of abiraterone with a prevalence of 17-30% in patients taking the medication [7]. There have been case reports of abiraterone-induced severe hypokalemia leading to morbidity and mortality, including torsades de pointes, seizures, and severe lethargy [10]. In a previous study, hypokalemia was noted in 16.6% of patients taking abiraterone and prednisone together [11], with an incidence of severe hypokalemia in 2.6-4.4% of patients [11,12]. Abiraterone-induced hypokalemia is managed with the use of exogenous glucocorticoids like prednisone. Exogenous glucocorticoids block ACTH release, re-establishing the negative feedback that was lost by reduced endogenous glucocorticoid production (Figure 1). The dosage of prednisone usually ranges from prednisone 5-10 mg daily in one or two divided doses. It is usually given with abiraterone from the start of therapy. Additionally, mineralocorticoid-receptor antagonists, such as spironolactone and eplerenone, help restore electrolyte balance by blocking mineralocorticoid receptors on nephrons and can be added if glucocorticoid supplementation is inadequate (Figure 2). Eplerenone is preferred to spironolactone due to spironolactone’s androgenic effect.

The timing of the onset of abiraterone-induced hypokalemia is not clear. Several case reports have shown varying durations for the onset of moderate to severe hypokalemia [13,14], including one month [10], and two weeks [10], after initiating therapy. In a case report where the patient did not adhere to routine potassium level checks, severe hypokalemia was seen 18 months after initiating abiraterone [15]. In our patient, the exact timing of the hypokalemia onset remained unclear due to his intermittent use of the medication. It is recommended to check serum potassium before the initiation of abiraterone and monthly thereafter.

Generally, the treatment of abiraterone-induced hypokalemia is usually by increasing the dose of prednisone, adding a mineralocorticoid receptor antagonist, minimizing potassium-depleting diuretics, and potassium supplementation. A double-blind, placebo-controlled, phase three trial of abiraterone demonstrated that abiraterone-induced hypokalemia be managed without the need to stop the medication, except in rare cases [16]. Fluid retention is also a common side effect of abiraterone, which was present in our patient. Workup for anasarca was negative for heart failure, renal failure, and liver failure. The patient had normal echocardiogram results, within-range BUN and creatinine levels, negative urinalysis for protein, and marginally elevated ALT, thereby establishing mineralocorticoid excess as the most probable cause.

**Figure 3:**
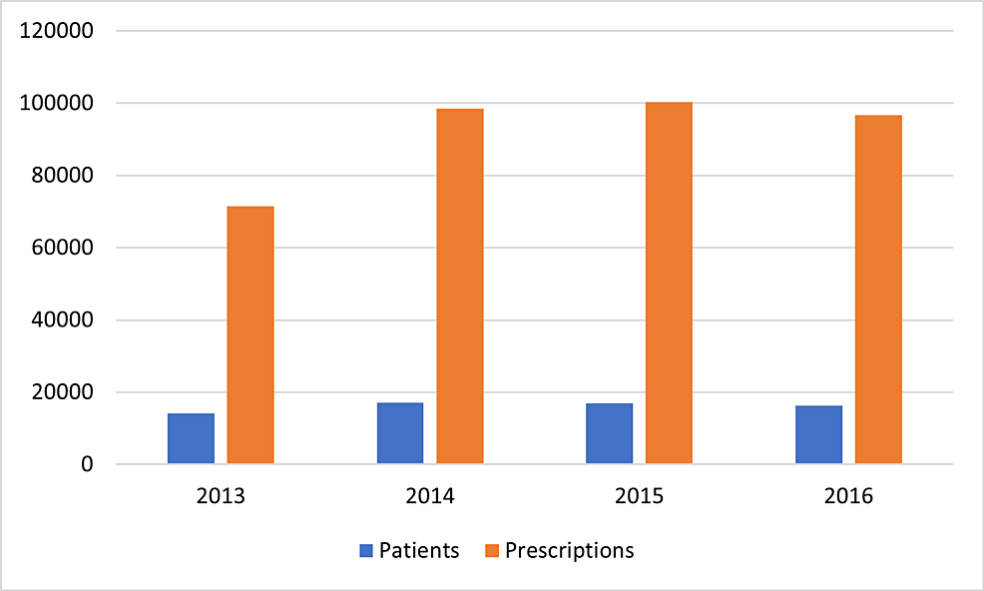
Abiraterone prescription rate between 2013 and 2016 Created with permission using data from Caram et. al, 2020 [8]

Put together, a deeper understanding of the timing and dose of prednisone, and mineralocorticoid-receptor antagonist initiation is necessary to achieve the best care for patients on abiraterone, as the rate of abiraterone prescription is on the increase (Figure 3). Currently, it is unclear if a glucocorticoid or mineralocorticoid receptor antagonist or a combination of glucocorticoid and mineralocorticoid receptor antagonists should be the preferred choice to control mineralocorticoid excess with abiraterone [14].

## Conclusions

Hypokalemia is a common adverse effect of abiraterone treatment. It occurs most times within the first year of therapy, sometimes as early as two weeks after initiation. Hypokalemia can lead to significant morbidity secondary to acute respiratory failure, muscle weakness, paresthesia, confusion, arrhythmias, constipation, polyuria, and polydipsia. Thus, it is recommended to check serum potassium prior to treatment and at least monthly while on abiraterone. It is also important to check serum ACTH and cortisol before the commencement of therapy and monitor for hypertension and fluid retention. With the increasing prevalence of prostate cancer and increasing prescriptions of abiraterone, healthcare providers need to be aware of the side effects associated with this medication and its monitoring and treatment, to minimize associated morbidity, mortality, hospitalizations, and associated healthcare costs.

## Figures and Tables

**Figure 4:**
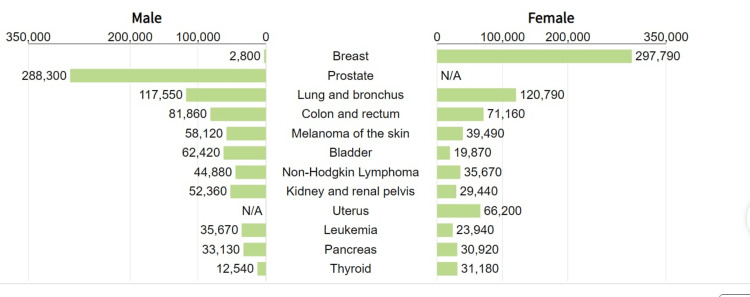
Projected cancer prevalence in the U.S. for the year 2023 Reused with permission from SEER Cancer Statistics Factsheets: Common Cancer Sites; National Cancer Institute [1]
